# Skin-to-Skin Care by Mother vs. Father for Preterm Neonatal Pain: A Randomized Control Trial (ENVIRON Trial)

**DOI:** 10.1155/2021/8886887

**Published:** 2021-01-04

**Authors:** Vivek Vishwanath Shukla, Anal Jitendrakumar Chaudhari, Somashekhar Marutirao Nimbalkar, Ajay Gajanan Phatak, Dipen Vasudev Patel, Archana Somashekhar Nimbalkar

**Affiliations:** ^1^Pramukhswami Medical College, Karamsad, Gujarat, India; ^2^University of Alabama at Birmingham, Birmingham, Alabama, USA; ^3^Central Research Services, Charutar Arogya Mandal, Karamsad, Gujarat, India

## Abstract

**Objective:**

To compare skin-to-skin care (SSC) given by mother and father for preterm neonatal pain control by premature infant pain profile (PIPP) score.

**Methods:**

64 stable preterm (28-36 weeks gestational age) neonates born at a level-3 neonatal intensive care unit were included in the trial. Random allocation with the help of a computer-generated sequence was done. In group A, SSC was given by the mother 15 minutes before the first heel-stick, and subsequently, SSC was given by the father before the second heel-stick. In group B, the sequence of SSC provider was reversed. Blinded PIPP score assessment at 0, 1, and 5 minutes of heel-stick were done by two independent assessors using video recording.

**Results:**

The mean (SD) birth weight was 1665.18 (339.35) grams, and mean (SD) gestational age was 34.28 (2.24) weeks. The PIPP score at 0, 1, and 5 minutes had no statistical or clinically significant differences between both groups (PIPP score mean (SD) at 0 minute = 3.20 (1.11) vs. 3.01 (1.29), *p* value = 0.38; 1 minute = 8.59 (4.27) vs. 8.26 (4.08), *p* value = 0.66; 5 minutes = 3.79 (1.40) vs. 3.93 (1.99), *p* value = 0.65 in SSC by mother and father group, respectively). Furthermore, there was no statistical difference between the groups for any components of the PIPP score (all *p* values > 0.05). The PIPP score at 5 minutes almost attained the 0-minute level in both the groups.

**Conclusion:**

Father is as effective as the mother for providing skin-to-skin care for preterm neonatal pain control. This trial is registered with CTRI/2018/01/016783.

## 1. Introduction

Preterm neonates are exposed to multiple painful procedures and interventions during their period of neonatal intensive care. The neonatal period is also the period of rapid brain maturation. Painful interventions during the neonatal period can cause irreversible changes in the developing brain and can negatively impact development [1]. Prematurity increases susceptibility for pain-induced neuronal injury and stress [2]. Neonatal intensive care also leads to decreased parental interactions and impacts parent-neonate bonding, which can also lead to poor neurodevelopmental outcomes [3]. Both pain and bonding issues can be addressed by providing pain control by skin-to-skin care by parents. Assessment of pain in preterm neonates is challenging [4]; however, premature infant pain profile (PIPP) is the most widely validated tool for pain assessment. Several pain control interventions are effective for preterm neonatal pain control [5], but SSC is one of the most preferred and widely studied pain control interventions because it confers several benefits in addition to pain control to both parents and neonates [6].

Often, the mother may not be available to provide SSC; in such cases, the father can be a feasible alternative to provide SSC. This can also help the father to get more involved in neonatal care, and it can provide the mother some time to convalesce from pregnancy and delivery. SSC provided by mother has been widely researched for preterm neonatal pain control [7–9]. SSC by mother can lead to multimodal stimulation, including olfactory [10, 11], auditory [12, 13], and tactile stimulation and thus confer pain control benefits. SSC by father has not been evaluated by such mechanistic studies, but the same mechanisms for pain control could also work when SSC is given by father. The comparative efficacy of SSC by father as compared to mother for preterm neonatal pain control has not been well studied [14]. The current blinded randomized crossover design trial is aimed at comparing the efficacy of SSC by mother and father for preterm neonatal pain control. We hypothesized that SSC by the father would result in comparable preterm pain control as that by the mother.

## 2. Methodology

The current trial was conducted from January 2018 to June 2019 at a university-affiliated level-3 NICU in Gujarat, India. The trial enrolled preterm neonates (28-36 weeks gestational age) admitted to the study NICU. Study interventions were performed on neonates expected to have a heel-stick procedure for point of care serum glucose assessment as per the protocol of the study NICU. Key exclusion criteria were neurological impairment (perinatal depression and hypoxic-ischemic encephalopathy ≥ stage 2 of Sarnat classification, grade 3/4 intraventricular hemorrhage, stroke, seizure, or congenital malformation), administration of analgesic/sedative medications (morphine, fentanyl, and midazolam) 24 hours before study interventions, neonatal abstinence syndrome, and critical illness (invasive mechanical ventilation and/or inotropes).

### 2.1. Study Design and Interventions

Randomization was performed using WINPEPI software by the statistician, and the assignment was placed in sealed opaque envelopes. Each participant's parent gave informed written consent to one of the resident physicians involved in the study before their enrolment. The neonatologist involved in the study opened the sealed opaque envelopes and allocated the SSC caregiver. Eligible participants were randomly assigned, in a 1 : 1 ratio, to group A or B. In group A participants, SSC was provided by the mother for 15 minutes before the first heel-stick and was continued postprocedure as per the protocol of the study institute. Subsequently, during the next heel-stick (6 or 12 hours after the first heel-stick as per the serum glucose monitoring requirement), SSC was provided by the father before heel-stick. In group B, the sequence was reversed. Video recording of the neonates' facial expression was done 1 minute before and 5 minutes after the procedure. PIPP scoring was done immediately before heel-stick (0 minute (baseline), 15 minutes after starting the SSC), at 1 minute after heel-stick and again at 5 minutes after heel-stick. In both groups, the neonates' face was turned to the side; care was taken to capture only the facial expression of the neonate without revealing SSC caregiver for blinded PIPP score assessment. A neonatology fellow and a pediatric resident trained in PIPP scoring and who had no involvement with participant allocation independently performed a blind PIPP score assessment based on the recorded videos. If the discrepancy was more than 1 point on the total PIPP score, it was resolved through discussion involving the senior neonatologist. Otherwise, the average of the PIPP scores was considered.

### 2.2. Sample Size

The standard deviation (SD) of the premature infant pain profile (PIPP) score was found to be about 4 from the previous studies in a similar setting [9, 15–17]. Considering a 2-point difference in the PIPP score as clinically important, a sample size of 64 per group was required at 5% alpha error and 80% power. Given the study design, we did not expect dropouts from the study.

### 2.3. Statistical Analysis

Descriptive statistics (mean (SD), frequency (%)) were used to depict the profile of the study participants. Independent sample *T* test was applied to compare the difference between the PIPP score between the groups at assessment time-points. An independent sample *T* test was used to evaluate the change in the PIPP score among the groups at 0 versus 5 minutes. The analysis was performed using STATA (14.1). The institutional ethics committee approved the trial protocol on 5 January 2018 via letter IEC/HMPCMCE/87/Faculty/14/03/18.

## 3. Results

**Figure 1:**
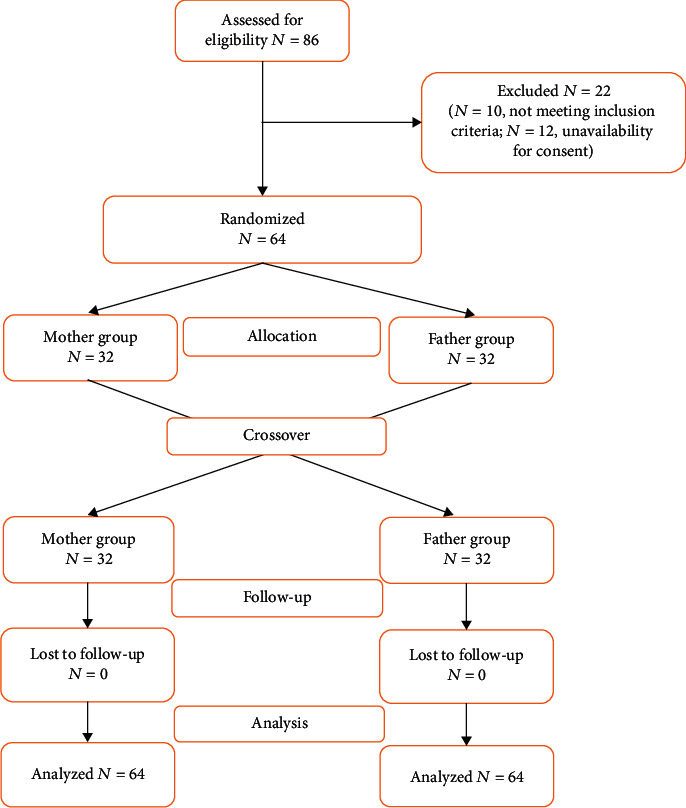
Participant flow diagram.

A total of 86 consecutive neonates admitted in the NICU were screened. Ten neonates did not meet the inclusion criteria, while fathers of 12 neonates did not give consent for the study. The refusal of consent was due to the unavailability of the fathers secondary to professional commitments. Finally, 64 neonates (females = 42%, males = 58%) were included in the study. No dropouts were encountered (Figure 1).

**Figure 2:**
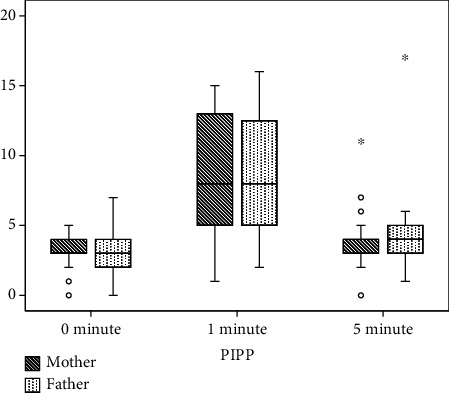
Group comparisons of PIPP scores at different time points. PIPP: premature infant pain profile score.

The mean (SD) birth weight and gestational age of the neonates were 1665.18 (339.35) grams and 34.28 (2.243) weeks, respectively. The mean (SD) age at the heel-stick of the neonates in both groups was similar. The PIPP score at 0, 1, and 5 minutes had no statistical or clinically significant differences between both groups (PIPP score mean (SD) at 0 minute = 3.20 (1.11) vs. 3.01 (1.29), *p* value = 0.38; 1 minute = 8.59 (4.27) vs. 8.26 (4.08), *p* value = 0.66; 5 minutes = 3.79 (1.40) vs. 3.93 (1.99), *p* value = 0.65 in SSC by mother and father groups, respectively) (Table 1). There was no statistically significant difference between the two groups for PIPP score components. The PIPP score at 5 minutes almost attained the 0-minute level in both the groups (Table 1 and Figure 2). The differences in PIPP scores between groups were neither statistically nor clinically significant. SSC was well tolerated in both the groups and no intervention-related adverse events were encountered.

## 4. Discussion

In the current trial, SSC by father was found to be equally effective for preterm neonatal pain control as compared to that of SSC by mother. There was no statistically significant difference between the two groups in all PIPP components. The result of the current trial is in contrast to a trial by Johnston et al. that had reported the pain control benefits of SSC by mother to be better than that by the father [14]. The key difference between the previous study and the current study is that the skin to skin duration was 30 minutes in the previous study, and they only followed the PIPP score till 2 minutes after the heel stick. In a literature review by the authors, this was the only study [14] comparing pain control effectiveness of SSC by mother and father.

SSC has been proven to confer several benefits in addition to pain control in preterm neonates [18–25], including several benefits to the mother [20–22], and improves parent-infant bonding [25]. Also, SSC has been established as an effective preterm neonatal pain control intervention [8, 17, 26–29], and so it is advocated as the most preferred preterm neonatal pain control intervention.

In some cases, the mother may not be available to provide SSC due to various postpartum complications. In such cases, the father can continue giving SSC while the mother recovers and later can promote SSC and can continue providing SSC as and when required [30].

Fatherhood is a unique experience marked by the aggregation of new roles and responsibilities, which might reduce father-neonate interaction and bonding. On the other hand, the mother is often left with the responsibility of caring for the newborn while she is convalescing from pregnancy and delivery. If the mother is unavailable to provide SSC, in such cases, the father can be a feasible alternative to provide SSC. This can also help the father to get more involved in neonatal care, and it can provide the mother some time to recover. Neonatal care can be made more family-oriented by encouraging fathers (in addition to the mother) to provide SSC [31]. In addition to pain control, as seen in the current trial, SSC by father has been shown to improve thermoregulation, neonatal behavior, and development [30, 31]. SSC by the father would provide an equal opportunity for the father for involvement in neonatal care, help in establishing bonding of the whole family, and promote equal parenthood.

The current trial is based on strong trial design. Randomized crossing over was chosen to reduce the impact of confounding factors. PIPP score assessments were made by two independent assessors who were blinded to the study assignment. The discrepancy of more than one point was addressed by discussion and assessment of the score by the senior author, who was also blinded to the intervention allocation. This methodology was adopted to eliminate potential assessment bias. The participation rate for the current trial was good, and there was no participant attrition. We were able to recruit the required number of participants to generate adequately powered results. The current study is the second trial to compare pain control efficacy of SSC by the father that to the mother and the first to show that the pain control efficacy is equal when either parent gives SSC.

Being a single-center study would be a limitation. We did not control prior illnesses and cumulative pain exposure before the study enrollment, which, although not well researched, could potentially impact the study result. We included preterm neonates from 28 to 36 weeks gestation to increase the generalizability of the results; however, the study was not powered to assess the differences in terms of outcomes for individual gestational ages.

## 5. Conclusion

Father is as effective as the mother for providing skin-to-skin care for preterm neonatal pain control. The current trial provides an additional basis for closer involvement of the fathers for participation in neonatal care. Further trials are needed to see whether the current trial findings are reproduced in term and extremely preterm (<28 weeks gestational age) neonates.

## Figures and Tables

**Table 1:** Comparison of PIPP scores by groups at various time points.

Variable	KMC by mother, *n* = 64, mean (SD)	KMC by father, *n* = 64, mean (SD)	*p* value
0 minute			
GA	0.67 (0.50)	0.67 (0.50)	Not applicable
BS	2.29 (0.84)	1.97 (1.07)	
HR	0.05 (0.21)	0.02 (0.13)	
OS	0 (0)	0 (0)	
BB	0.13 (0.33)	0.20 (0.41)	
ES	0.03 (0.18)	0.09 (0.29)	
NF	0.03 (0.18)	0.06 (0.24)	
PIPP	3.20 (1.12)	3.02 (1.29)	
1 minute			
GA	0.67 (0.51)	0.67 (0.51)	>0.999
BS	2.05 (0.70)	1.81 (0.77)	0.08
HR	1.23 (0.53)	1.14 (0.66)	0.38
OS	0.16 (0.37)	0.11 (0.31)	0.44
BB	1.53 (1.21)	1.53 (1.14)	>0.999
ES	1.47 (1.26)	1.52 (1.15)	0.83
NF	1.48 (1.26)	1.48 (1.15)	>0.999
PIPP	8.59 (4.27)	8.27 (4.08)	0.66
5 minutes			
GA	0.67 (0.51)	0.67 (0.51)	>0.999
BS	2.28 (0.79)	2.09 (0.87)	0.20
HR	0.28 (0.45)	0.34 (0.60)	0.51
OS	0 (0)	0.03 (0.25)	0.32
BB	0.28 (0.58)	0.38 (0.58)	0.36
ES	0.14 (0.47)	0.20 (0.51)	0.47
NF	0.14 (0.47)	0.22 (0.52)	0.37
PIPP	3.80 (1.40)	3.94 (1.99)	0.65

PIPP: premature infant pain profile; GA: gestational age; BS: behavioral state; HR: heart rate; OS: oxygen saturation; BB: brow bulge; ES: eye squeeze; NF: nasolabial fold.

## Data Availability

The trial data used to support the findings of this study are available from the corresponding author upon request.
